# Serologic evaluation of cytomegalovirus (CMV), Toxoplasma gondii and Brucella in schizophrenia patients 

**DOI:** 10.22088/cjim.14.3.560

**Published:** 2023

**Authors:** Seyed Shahab Banihashem, Forough Yousefi Saber, Somayeh Motazedian, Masoud Mardani, Alireza Shamsi, Maryam Nazari, Nastaran Samani, Arash Danesh

**Affiliations:** 1 Department of Psychosomatic Medicine, Taleghani Hospital, School of Medicine, Shahid Beheshti University of Medical Sciences, Tehran, Iran; 2 Department of Psychiatry, Taleghani Hospital, School of Medicine, Shahid Beheshti University of Medical Sciences, Tehran, Iran; 3 Infectious Disease and Tropical Medicine Department, Faculty of Medicine of Shaheed Beheshti University of Medical Sciences and Health Services, Tehran, Iran; 4 Department of Neurology, School of Medicine, Shahid Beheshti University of Medical Sciences, Tehran, Iran

**Keywords:** Schizophrenia, Serology, Cytomegalovirus, Brucellosis, Toxoplasmosis

## Abstract

**Background:**

Infectious agents are considered as a possible cause of schizophrenia. The aim of this study was to evaluate the serum levels of cytomegalovirus (CMV), Toxoplasma gondii and Brucella antibodies in schizophrenia patients compared with the control group.

**Methods:**

This cross-sectional study was performed on 75 patients with schizophrenia who were clinically diagnosed with schizophrenia using the Diagnostic and Statistical Manual of Mental Disorders (DSM-5) by two independent psychiatrists. As the controls, 75 sex and age-matched individuals were selected from orthopedic and surgical wards, who were admitted because of trauma. Anti-Toxoplasma gondii IgG antibody was detected by Abbott's company diagnostic kit. To detect anti-Brucella IgG antibodies, the enzyme-linked immunosorbent assay (ELISA) test with Vircell diagnostic kit was used. Quantitative luminescence (CLIA) method using Abbott diagnostic kit was also used to detect anti-cytomegalovirus IgG antibody (CMV IgG avidity).

**Results:**

There was not any clinically significant differences in the mean value of Toxoplasma, CMV and Brucella IgG antibodies between schizophrenia and control group. However, considering cut-off point for these tests and further analysis with non-parametric tests showed clinically significant difference between two groups at cut-off point 1.1 for anti-Brucella IgG antibody which indicated more positive samples in schizophrenia group (24 out of 75) than control group (12 out of 75) with a p-value less than 0.05 (0.046).

**Conclusion:**

The results of the present study showed no association between toxoplasmosis infection and CMV and schizophrenia. However, there might be a positive correlation between anti-Brucella IgG antibody and schizophrenia.

Among all brain diseases, schizophrenia is one of the most disabling ones with a life time prevalence of 0.6-1.9 %. This condition presented with severe and constant psychotic and cognitive problems (1). In addition to some genetic factors, many environmental factors might be involved, such as exposure to microorganisms or prenatal malnutrition, birth defects, and other psychological factors (2). Immunological abnormalities are also considered to be related with schizophrenia. This correlation would be explained by either primary autoimmune disorders or the effects of neurotoxic viruses. Well-designed research for detecting any association neurotoxic viral infections and schizophrenia showed negative results; however epidemiological data indicated a high incidence of schizophrenia after prenatal exposure to influenza during several viral epidemics. There are some other data that support a viral hypothesis including high number of congenital anomalies, increased rate of pregnancy and birth complications, seasonality of birth align with viral infection, geographical clusters of adult cases, and seasonality of hospitalizations (3-5).

What is going on in the pathophysiology of neurodegenerative and neurobehavioral diseases might be explained by the effects of different infectious organisms such as infected macrophages of the brain by these organisms or crossing the blood-brain barrier and transmission into the peripheral nerves (6, 7). The prevalence of Toxoplasma gondii infection is around 30% (8).

 Its correlation with schizophrenia has been shown in some studies. A meta-analysis by Torrey et al. of 38 studies showed that individuals with schizophrenia have an increased prevalence of T. gondii antibodies (9); However, there has been no association between schizophrenia and toxoplasmosis in some other studies (10-12). Another recent meta-analysis reported that there might be a correlation between reactivation of Toxoplasma gondii infection and schizophrenia as well as other psychiatric disorders (13). Several studies in various parts of Iran were done to identify the association of schizophrenia with T. gondii with both positive and negative results (14). 

Brucellosis is the most prevalent bacterial zoonosis in the world, affecting more than 500,000 new cases annually (15, 16). Brucellosis can cause Neurobrucellosis as a rare but serious complication with various clinical manifestation as a result of affecting central or peripheral nervous system (17-20). 

Although psychosis is one of relatively rare presentation of neurobrucellosis, there have been some case reports of brucellar psychosis (20, 21). Cytomegalovirus (CMV) is a prevalent viral pathogen, and in most of cases the primary infection is in-apparent (22). 

It has been shown in literature that there is an elevated level of CMV antibody in patients with recent onset schizophrenia (23). In addition, CMV infection may exacerbate symptoms in patients with schizophrenia (24). Considering schizophrenia as a pervasive, relatively common and very debilitating psychiatric disorder of unknown etiology, and also conflicting data about possible causal correlation between infectious agents and schizophrenia, we decided to evaluate the serum levels of cytomegalovirus (CMV), Toxoplasma gondii and brucellosis antibodies in patients with schizophrenia compared with the control group. Additionally, with regard to the significance of geographical variation in the prevalence of infectious disease and schizophrenia, it was needed to design this seroprevalence study in Iranian population, and as far as we know, this is the first case-control study in this population to evaluate all three abovementioned organisms at the same time in schizophrenia patients.

## Methods

**Study population: **This study was performed on 75 patients with schizophrenia, diagnosed by two independent psychiatrists using the Diagnostic and Statistical Manual of Mental Disorders, Fifth Edition (DSM-5). The inclusion criteria was the final diagnosis of schizophrenia disorder, and the exclusion criteria was history of brucellosis. Sample size was based on available cases between August 2016 to August 2017.

**Controls: **Control group comprised of 75 patients from orthopedic and surgical wards, who were admitted because of trauma, and were sex and age-matched with the case group. The inclusion criteria for the control group was admitting due to trauma (orthopedic or surgical), and the exclusion criteria included a history of mental illness and any underlying disease (especially a history of brucellosis or any complaints of fever, chills and long-term joint pain in recent months).

**Overall design: **In this case-control cross-sectional study, both case and control groups were selected from patients admitted in Taleghani Hospital in Tehran, Iran, from August 2016 to August 2017. For all the identified individuals, collected blood samples were transferred to the library for immunological assays. It should be mentioned that with using a coded serum sample, the laboratory was blind about case and control samples. The sera of the subjects were kept at -20 °C until the experiment.

**Immunological assays: **Plasma samples from all participants were initially collected in 5 ml plasma preparation tubes, centrifuged and frozen within 6 hours after blood collection. The 150 selected plasma samples from cases and controls were transferred to the Noor Laboratory for analysis of specific enzyme-based immunoassays for immunoglobulin (IgG) class antibodies against Toxoplasma gondii, CMV, and Brucella.

***Toxoplasma gondii: *** Anti- Toxoplasma gondii IgG antibody was detected by Abbott's company diagnostic kit. Abbott Imx uses an automated system based on microparticle enzyme immunoassay technology to detect Toxoplasma gondii antibodies. According to the manufacturer's criteria, interpretation of IgG IMx results were as follows: more than 3.0 IU/ml for IgG considered as positive antibodies, between 2.0 and 3.0 IU/ml as suspicious result, and less than 2.0 IU/ml as negative for IgG antibodies. 

***Brucella:*** To detect anti-Brucella IgG antibodies, enzyme-linked immunosorbent assay (ELISA) test with Vircell diagnostic kit was used. In this regard, values greater than or equal to 0.1 were considered as positive test results, and values less than 0.9 were considered as negative test results. 

***CMV: *** Quantitative luminescence (CLIA) method using Abbott diagnostic kit was also used to detect anti-cytomegalovirus IgG antibody (CMV IgG avidity) in the samples. According to the manufacturer's instructions, interpretation of results was as follows: the values less than or equal to 0.2 considered as low avidity, ranged 0.21 to 0.30 as medium avidity, and more than 0.30 as high avidity. In particular, avidity less than 0.2 was an indicator of the possibility of primary infection in less than 3 months before sample collection.

**Statistical methods: **After sample-analysis and quality control, statistical analysis was conducted on 150 cases and controls. Descriptive statistics such as the mean, median, percentage, frequency distribution and standard deviation, and analytical statistics such as one-way analysis of variance (ANOVA), chi-squire and Pearson's Correlation Coefficient were used for analyzing the results. Odds ratios (ORs) and 95% likelihood ratio confidence intervals (95% CIs) were calculated. A two-sided p value of less than 0.05 was considered to be statistically significant. Statistical analyses were performed using the SPSS Version IBM 21.

**Ethics: **The Ethics Committee of Shahid-Beheshti University of Medical Sciences approved the study on 21.04.2021 (IR.SBMU.RETECH.REC.1400.039). This committee also approved the transport and analysis of plasma samples to the Noor Laboratory. The purpose of study was explained to the patients and control group, and written informed consent was obtained from them, and all participants’ names remained confidential.

## Results

75 patients with schizophrenia disorder and 75 healthy controls who were age and sex-matched were enrolled. Each group included 48 men and 27 women (64% and 36%, respectively). The mean age of participants at case and control groups were 43.23 ± 14.90 years and 43.51 ± 14.40 years, respectively (P = 0.889). The mean value of Toxoplasma IgG in schizophrenia and control groups were respectively 8.85 ± 14.6 and 9.64 ± 24.38, indicating no clinically significant difference (P= 0.811, F=0.057). In addition, the mean value of cytomegalovirus IgG antibody in two groups were respectively 198.64 ± 62.81 and 212.70 ± 56.06, with no clinically significant difference (P= 0.150, F=2.092). 

Finally, the mean value of anti-Brucella IgG antibody in two groups were respectively 1.88 ± 3.31 and 1.45±2.42, with no clinically significant difference (P= 0.361, F=0.838). As a result, the comparison of mean value of Toxoplasma, CMV and Brucella antibodies between case and control groups with one-way analysis of variance indicated no clinically significant difference (table 1).

Considering the cut-off point for these tests and further analysis with non-parametric tests such as chi-squire showed no clinically significant difference between two groups apart from cut-off point 1.1 for anti-Brucella IgG antibody which indicated more positive samples in schizophrenia group (24 out of 75) than control group (12 out of 75) with a p-value of 0.046 (table 2). The mean value analysis of antibodies between two groups based on sex with one-way ANOVA and Welch tests showed higher index for anti-CMV IgG antibody in female (F (1.73) =7.457, P = 0.007 and W (1.71) = 10.623, P = 0.005). In addition, when in schizophrenia group, the mean value of antibodies based on age was analyzed using Pearson's Correlation Coefficient, a positive result for anti-CMV IgG antibody was revealed (the higher age, the more positive results, P = 0.001). 

**Table 1:** One-way ANOVA results regarding the comparison of mean value of Toxoplasma, CMV and Brucella IgG antibodies between case and control groups

**P-value**	**F**	**Mean square**	**df**	**Sum squire**	**Source of variance**	**The mean value of antibodies**
0.811	0.057	23.128	1	23.128	Between group	**Toxoplasma**
		403.918	148	59779.843	Within group	
0.150	2.092	7415.947	1	7415.947	Between group	**CMV**
		3544.512	148	524587.749	Within group	
0.361	0.838	7.059	1	7.059	Between group	**Brucella**
		8.424	148	1246.790	Within group	

**Table 2:** Chi-squire results regarding the comparison of positive and negative results of Toxoplasma, CMV and Brucella IgG antibodies between case and control groups

**variants**	**Group**	**Positive**		**Negative**		**P-value**
		**Frequency**	**Percent**	**Frequency**	**Percent**	
**Toxoplasma**	Case	33	56	42	44	0.414
	Control	38	50.70	37	49.30	
**CMV**	Case	69	92	6	8	0.513
	Control	71	94.70	4	5.30	
**Brucella 1.1**	Case	36	48	39	52	0.064
	Control	24	32	51	68	
**Brucella 1.8**	Case Control	12 12	16 16	63 63	84 84	1.000

## Discussion

In our study, there was no statistically significant difference between the mean value of Toxoplasma, CMV and Brucella IgG antibodies between case and control groups. However, considering cut-off point for serum levels of these antibodies, positive results of anti-Brucella IgG antibody in schizophrenia group (24 out of 75) were more than control group (12 out of 75) with a p-value of 0.046. In most studies, contrary to the results of the present study, the prevalence of Toxoplasma gondii in patients with schizophrenia was significantly higher than controls (25-27), and in a small number of them, no significant difference was seen (28). 

These contradictory results might be partly due to control groups’ profile differences in Toxoplasma related studies. The same as or study results, there are some other studies with negative association between schizophrenia and Toxoplasma gondii seropositivity; In Campos-Carli et al.’s study, the prevalence and titers of *T. gondii *IgM and IgG antibodies did not differ between patients and controls (29). 

Furthermore, this negative correlation has also been found in Iranian studies in which Saraei-Sahnesaraei et al. (30) and Daryani et al.’s studies (31) did not find any significant association, which is contrary to the finding of other Iranian studies; For example, Hamidinejat et al. (32), Alipour et al. (33), Ansari-Lari et al. (34) and Abdollahian et al. (35) found significantly higher seropositive rates among patients with schizophrenia. Toxoplasma gondii genome might be one possible reason for these conflicting results. 

This protozoan has genotypes with significant differences in virulence and geographical distribution. In addition, various variables of patients and controls must be peer-to-peer matched including age, socio-economic status and geographical location. However, it has not been a confounding factor in our study. Based on research, some neuropsychiatric diseases including schizophrenia might be related to latent toxoplasmosis (36). 

In this regard, Fond et al.’s study indicated that there was a significant positive correlation between latent Toxoplasma infection and schizophrenia patients (37) .In addition, another meta-analysis reported the association of toxoplasmosis reactivation with several psychiatric disorders, especially schizophrenia (38). It is worth mentioning that this possible causal correlation is not specific for schizophrenia, and this organism might be related to other psychiatric problems. For example, its association with schizophrenia and bipolar disease has been shown in a recently published meta-analysis (39). This correlation is considered to be causal as in a large-scale study by Burgdorfa et al. on 81,912 individuals, has been indicated that T. gondii infection might be a possible causal factor for schizophrenia (24).

Based on some studies, it is highly possible that the limbic system gets infected with CMV which has a strong propensity to invade the nervous system (40). Our study showed that the higher serum level of cytomegalovirus IgG antibody in the case group was not statistically significant. Various studies have been designed to investigate the relationship between CMV and schizophrenia. Srikanth et al. found a significant temporal correlation between elevated CMV antibody levels and new onset psychosis (41. Furthermore, Shirt et al.’s study on 329 patients with schizophrenia showed that positive serological results for CMV were associated with cognitive impairment in schizophrenia (42). 

Finally, a recent Iranian study in 2016 indicated higher levels of CMV IgG antibody in schizophrenia and bipolar patients, in comparison to healthy control group (43). However, studies before 1992 did not show any association between CMV antibody levels and schizophrenia which might be a result of using the less sensitive assays and unmatched controls (23).The negative correlation result between anti-CMV IgG antibody and schizophrenia in this study is in contrast to other studies, and it might be due to different geographical distribution, different immunoassay methods and tests validity, and also variability in sample size and participants' variables. 

Human brucellosis is an endemic disease in some part of the world including the Middle East. A recent meta-analysis in Iran by Mirnejad et al. indicated that the pooled incidence of brucellosis was 0.001% annually, and based on geographical distribution, its relative frequency varied from 7.0/100000 to 276.41/100000 (44). Neurobrucellosis occurs in 5–10% of cases of brucellosis. However psychiatric presentations in neurobrucellosis are rare, there are few reports of Brucella induced psychosis in the literature (45-47).

 In an Iranian study carried out by Shoaei et al., positive serologic results for Brucella in 500 hospitalized psychiatric patients were higher than non-psychiatric patients (48). There have also been some case report articles about neurobrucellosis; For example, Bidaki and Sheibani reported some neurobrucellosis cases presenting with psychotic symptoms (49, 50). In addition, Kilik et al. reported a 27-year-old patient presented with acute psychotic disorder, but finally received neurobrucellosis diagnosis (51). These observations indicate that in patients who experience unexplained neurological and atypical psychotic symptoms, neurobrucellosis should be considered as a differential diagnosis especially in endemic regions such as Iran. 

In conclusion, the results of the present study showed no clinically significant difference between the mean value of Toxoplasma, CMV and Brucella antibodies in schizophrenic patients and control group. However, considering a cut-off point, revealed a clinically significant correlation between more positive results for anti-Brucella IgG antibody and schizophrenia (schizophrenic patients had more positive results of anti-Brucella antibody than control group).

 Therefore, it is thought that long prospective or retrospective cohort and involving more cases are needed to evaluate any cause and effect correlation between Brucella infection and developing schizophrenia disorder. For example, since almost all of brucellosis cases in Iran have been reported and recorded over the past 40 years, a retrospective cohort could be designed to examine whether exposure to brucellosis in pregnancy or early childhood is associated with schizophrenia outcome in adulthood or not.

## References

[1] Tammainga CA, Sadock B (2017). Schizophrenia and other psychotic disorders. Kaplan and Sadock’s comprehensive textbook of psychiatry.

[2] Nicolson GL, Haier J (2009). Role of chronic bacterial and viral infections in neurodegenerative, neurobehavioral, psychiatric, autoimmune and fatiguing illnesses: Part 1. Br J Gen Pract.

[3] Sadock BJ, Sadock VA, Ruiz P (2019). Schizophrenia spectrum and other psychotic disorders:Synapsis of psychiatry Behavioral sciences/Clinical psychiatry.

[4] McGlashan TH, Hoffman RE (2000). Schizophrenia as a disorder of developmentally reduced synaptic connectivity. Arch Gen Psychiatry.

[5] Kim JJ (2007). Viral infections as etiological factors of schizophrenia. Psychiatry Investig.

[6] Dickerson F, Severance E, Yolken R (2017). The microbiome, immunity, and schizophrenia and bipolar disorder. Brain BehavImmun.

[7] Prasadarao NV, Wass CA, Weiser JN (1996). Outer membrane protein A of Escherichia coli contributes to invasion of brain microvascular endothelial cells. InfectImmun.

[8] Halonen SK, Weiss LM (2013). Toxoplasmosis. HandbClinNeurol.

[9] Torrey EF, Bartko JJ, Yolken RH (2012). Toxoplasma gondii and other risk factors for schizophrenia: an update. Schizophr Bull.

[10] De Witte LD, Van Mierlo HC, Litjens M (2015). The association between antibodies to neurotropic pathogens and schizophrenia: a case-control study. NPJ Schizophr.

[11] Karabulut N, Bilgiç S, Gürok MG, Karaboğa F (2015). Is there any role of latent toxoplasmosis in schizophrenia disease?. J Chin Med Assoc.

[12] Sugden K, Moffitt TE, Pinto L (2016). Is Toxoplasma gondii infection related to brain and behavior impairments in humans? Evidence from a population- representative birth cohort. PLoSOne.

[13] Sutterland AL, Fond G, Kuin A (2015). Beyond the association Toxoplasma gondii in schizophrenia, bipolar disorder, and addiction: systematic review and meta-analysis. ActaPsychiatrScand.

[14] Ansari-Lari M, Farashbandi H, Mohammadi F (2017). Association of Toxoplasma gondii infection with schizophrenia and its relationship with suicide attempts in these patients. Trop Med Int Health.

[15] Buzgan T, Karahocagil MK, Irmak H (2010). Clinical manifestations and complications in 1028 cases of brucellosis: A retrospective evaluation and review of the literature. Int J Infect Dis.

[16] Hatami H, Saghari H, Hatami H ( 2005). Epidemiology of Brucellosis. Epidemiology and control of diseases related to Bioterrorism.

[17] Eren S, Bayam G, Ergönül Ö (2006). Cognitive and emotional changes in neurobrucellosis. J Infect.

[18] Mandel GL, Bennett JE, Dolin R (2010). Bennett’s principles and practice of infectious diseases. 7th ed. Philadelphia, PA: Churchil Livingstone Elsevier.

[19] Shehata GA, Abdel-Baky L, Rashed H, ElaminH (2010). Neuropsychiatric evaluation of patients with brucellosis. J Neurovirol.

[20] Ghaffarinejad AR, Sarafzadeh F, Sedighi B, Sadeghieh T (2008). Psychosis as an Early Presentation of Neuro-Brucellosis. Iran J Med Sci.

[21] Ates MA, Algül A, Geçc Ö (2008). Acute psychosis due to neurobrucellosis: a case report. Anatolian J psychiatry.

[22] Taylor GH (2003). Cytomegalovirus. Am Fam Physician.

[23] Torrey EF, Leweke MF, Schwarz MJ (2006). Cytomegalovirus and schizophrenia. CNS Drugs.

[24] Burgdorf KS, Trabjerg BB, Pedersen MG (2019). Large-scale study of toxoplasma and cytomegalovirus shows an association between infection and serious psychiatric disorders. Brain BehavImmun.

[25] Wang HL, Wang GH, Li QY (2006). Prevalence of Toxoplasma infection in first‐episode schizophrenia and comparison between Toxoplasma‐seropositive and Toxoplasma‐seronegative schizophrenia. ActaPsychiatrScand.

[26] Cetinkaya Z, Yazar S, Gecici O, Namli MN (2007). Anti-Toxoplasma gondii antibodies in patients with schizophrenia—preliminary findings in a Turkish sample. SchizophrBull.

[27] Chen X, Chen B, Hou X (2019). Association between toxoplasma gondii infection and psychiatric disorders in Zhejiang, Southeastern China. Acta Trop.

[28] Amminger GP, McGorry PD, Berger GE (2007). Antibodies to infectious agents in individuals at ultra-high risk for psychosis. Biol Psychiatry.

[29] Campos-Carli SM, Marciano Vieira EM, Pessoa Rocha N (2017). Toxoplasma gondii infection and chronic schizophrenia: is there any association?. Arch Clin Psychiatry.

[30] Saraei-Sahnesaraei M, Shamloo F, JahaniHashemi H, Khabbaz F, Alizadeh S Relation between Toxoplasma gondii infections and schizophrenia. Iran J Psychiatry ClinPsychol2009.

[31] Daryani A, Sharif SM, Hosseini SH, Karimi SA, Gholami S (2010). Serological survey of Toxoplasma gondii in schizophrenia patients referred to Psychiatric Hospital, Sari City, Iran. Trop Biomed.

[32] Hamidinejat H, Ghorbanpoor M, Hosseini H (2010). Toxoplasma gondii infection in first-episode and inpatient individuals with schizophrenia. Int J Infect Dis.

[33] Alipour A, Shojaee S, Mohebali M (2011). Toxoplasma infection in schizophrenia patients: a comparative study with control group. Iran J Parasitol.

[34] Ansari-Lari M, Farashbandi H, Mohammadi F (2017). Association of Toxoplasma gondii infection with schizophrenia and its relationship with suicide attempts in these patients. TropMedIntHealth.

[35] Abdollahian E, Shafiei R, Mokhber N, Kalantar K, Fata A (2017). Seroepidemiological study of toxoplasma gondii infection among psychiatric patients in Mashhad. Iran J Parasitol.

[36] Fabiani S, Pinto B, Bonuccelli U, Bruschi F (2015). Neurobiological studies on the relationship between toxoplasmosis and neuropsychiatric diseases. J NeurolSci.

[37] Fond G, Boyer L, Schürhoff F (2018). Latent toxoplasma infection in real-world schizophrenia: Results from the national FACE-SZ cohort. Schizophr Res.

[38] Sutterland AL, Fond G, Kuin A (2015). Beyond the association Toxoplasma gondii in schizophrenia, bipolar disorder, and addiction: systematic review and meta-analysis. ActaPsychiatrScand.

[39] Vlatkovic S, Sagud M, SvobStrac D (2018). Increased prevalence of toxoplasma gondiiseropositivity in patients with treatment-resistant schizophrenia. Schizophr Res.

[40] Kenneson A, Cannon MJ (2007). Review and meta‐analysis of the epidemiology of congenital cytomegalovirus (CMV) infection. Rev Med Virol.

[41] Srikanth S, Ravi V, Poornima KS (1994). Viral antibodies in recent onset, nonorganic psychoses: correspondence with symptomatic severity. Biol Psychiatry.

[42] Shirts BH, Prasad KM, Pogue-Geile MF (2008). Antibodies to cytomegalovirus and herpes simplex virus 1 associated with cognitive function in schizophrenia. Schizophr Res.

[43] SuleimaniMohammadi F, RahimiForoushani A, Rokni M, Farahmand M, Ahmadi Kia K, Shadab A, Ahmadkhaniha H, Yavarian J (2017). Comparative study of cytomegalovirus antibody and viral load in schizophrenia and bipolar patients. Tehran University Medical Journal TUMS Publications..

[44] Mirnejad R, MasjedianJaziF, MostafaeiSH, Sedighi M (2017). Epidemiology of brucellosis in Iran: A comprehensive systematic review and meta-analysis study. MicrobPathog.

[45] Eren S, Bayam G, Ergönül Ö (2006). Cognitive and emotional changes in neurobrucellosis. J Infect.

[46] Karsen H, Akdeniz H, Karahocagil MK, Irmak H, SünnetçioğluM (2007). Toxic-febrile neurobrucellosis, clinical findings and outcome of treatment of four cases based on our experience. Scand J Infect Dis.

[47] Eren S, Bayam G, ErgonulOC¸Elikbas A (2006). Cognitive and emotional changes in neurobrucellosis. J Infect.

[48] Shoaei SD, Bidi N (2012). Serologic evaluation of brucellosis in patients with psychiatric disorders. Caspian J Intern Med.

[49] Bidaki R, Yassini SM, Maymand MT (2013). Acute psychosis due to brucellosis in a rural Iran: a report of two cases. J Inter Infect Microbial.

[50] Sheibani F, Sarvghad M R, Bojdi A, Naderi HR (2017). Brucellarpsychosis. ArchIranMed.

[51] Kilic F, NurluTemel E, Sevicin L, Ayaz F (2019). Patient with acute psychosis due to neurobrucellosis: a rare case. Psychiatry BehavSci.

